# The BlueBio project’s database: web-mapping cooperation to create value for the Blue Bioeconomy

**DOI:** 10.1038/s41597-023-02078-2

**Published:** 2023-04-19

**Authors:** Anna Nora Tassetti, Carmen Ferrà, Tania Manarini, Fabrizio Moro, Massimiliano Pinat, Jacopo Pulcinella, Elisa Punzo, Vera Salvalaggio, Paolo Scarpini, Alessandra Spagnolo, Kristin Elisabeth Thorud, Gianna Fabi

**Affiliations:** 1 National Research Council, Institute for Marine Biological Resources and Biotechnologies (CNR-IRBIM), Ancona, Italy; 2 NBFC, National Biodiversity Future Center, Palermo, Italy; 3 Department of Biological Geological and Environmental Sciences (BiGeA), University of Bologna, Bologna, Italy; 4 Almawave SpA, Rome, Italy; 5 Research Council of Norway (RCN), Oslo, Norway

**Keywords:** Databases, Funding, Marine biology, Research management, Institutions

## Abstract

Funding innovation requires knowledge on previous/on-going research and identification of gaps and synergies among actors, networks and projects, but targeted databases remain scattered, incomplete and scarcely searchable. Here we present the BlueBio database: a first comprehensive and robust compilation of internationally and nationally funded research projects active in the years 2003–2019 in Fisheries, Aquaculture, Seafood Processing and Marine Biotechnology. Based on the previous research projects’ database realized in the framework of the COFASP ERA-NET, it was implemented within the ERA-NET Cofund BlueBio project through a 4-years data collection including 4 surveys and a wide data retrieval. After being integrated, data were harmonised, shared as open and disseminated through a WebGIS that was key for data entry, update and validation. The database consists of 3,254 “georeferenced” projects, described by 22 parameters that are clustered into textual and spatial, some directly collected while others deduced. The database is a living archive to inform actors of the Blue Bioeconomy sector in a period of rapid transformations and research needs and is freely available at: 10.6084/m9.figshare.21507837.v3.

## Background & Summary

Knowing previous and on-going research and identifying gaps and synergies among actors, networks and projects is key to both target and fund successful innovation. Nevertheless, related information remains scattered and incomplete, as well as dedicated databases are scarcely searchable.

In the last decades, several databases of research projects have been implemented, either under the promotion of the European Union, e.g. CORDIS^1^ and Keep.eu^2^, as well as by research institutions and funding organisations, e.g. Matís^3^ (Iceland), National Marine Fisheries Research Institute^4^ (Poland), Norwegian Seafood Research Fund - FHF^5^, Foundation for Science and Technology - FCT^6^ (Portugal), but they suffer from several gaps that limit their usefulness for delivering information that can be easily downloaded, aggregated and interpreted.

Most importantly, usually these databases do not include both projects funded at international and national levels, and - even when they do - they are limited to projects involving specific actors (e.g., the government-owned non-profit research company Matís from Iceland) or specific countries (e.g., Norway in FHF-financed R&D projects). Last but not least, most of these research projects’ databases do not provide georeferenced information at the level of single projects.

Here we introduce the BlueBio database, as a first comprehensive and harmonised compilation of research projects funded at transnational and national level by the EU Member States (MS), the European Commission and other funding programmes and initiatives (e.g., bilateral/multilateral Cooperation Agreements) in Fisheries, Aquaculture, Seafood Processing and Marine Biotechnology.

The database was generated within the ERA-NET Cofund BlueBio (2019–2024) “Unlocking the Potential of Aquatic Bioresources”^7^, funded within the EU Horizon 2020 programme. It involves 30 partners (i.e., funding agencies) from 17 countries and builds on a collaboration between JPI Oceans and the previous ERA-NETS COFASP (Cooperation in Fisheries, Aquaculture and Seafood Processing)^8^ and ERA-MBT (MarineBiotech)^9^ funded within the EU 7^th^ Framework Programme (FP7).

The ERA-NET scheme was launched by the EU in 2002 to support the coordination and collaboration between national and regional research programmes and to increase the share of funding that Member States jointly dedicate to challenge-driven research and innovation agendas. The focus and role of ERA-NETs have varied across the Frameworks Programmes:

- ERA-NET actions in FP6 provided support for actors implementing public research programmes to coordinate their activities, e.g. by developing joint activities such as joint calls for transnational proposals;
- ERA-NET Plus actions in FP7 provided - in a limited number of cases with high European added value - additional EU financial support to top-up research funding of a single joint call for proposals between national and/or regional programmes;
- The ERA-NET Cofund under Horizon 2020 merged the former ERA-NET and ERA-NET Plus into a single instrument with the central and compulsory element of implementing one substantial call with top-up funding from the Commission.

The main objective of the BlueBio project is to establish a coordinated R&D funding scheme that will strengthen Europe’s position in the blue bioeconomy, identifying new and improving existing ways of bringing bio-based products and services to the market and finding new ways of creating value from the blue bioeconomy. In this process a key issue is the development of a future research agenda in the field of marine biotechnologies associated to fisheries, aquaculture and seafood processing, based on the past, current and future challenges posed to research. To achieve this objective, a focal activity was the analysis of the past and on-going research projects on aquaculture, fisheries and seafood processing funded at European/national/regional level, including an identification of possible duplications and gaps.

The PostgreSQL BlueBio database was built on the basis of the previous COFASP database^10,11^ of research projects on Fisheries, Aquaculture, Seafood Processing (FASP) active in the period 2003–2013, by adding international/national research FASP projects funded since 2014 and projects dealing with Marine Biotechnology (active in the period 2003–2019). Hence the database gathers projects further beyond the ERA-NET scheme. The analysis of the collected information allowed us to cluster projects into research topics and to list those that would need to be further investigated in the short-medium period and in the different EU areas^12^. It could be pivotal to good policy making and development^10^.

**Fig. 1:**
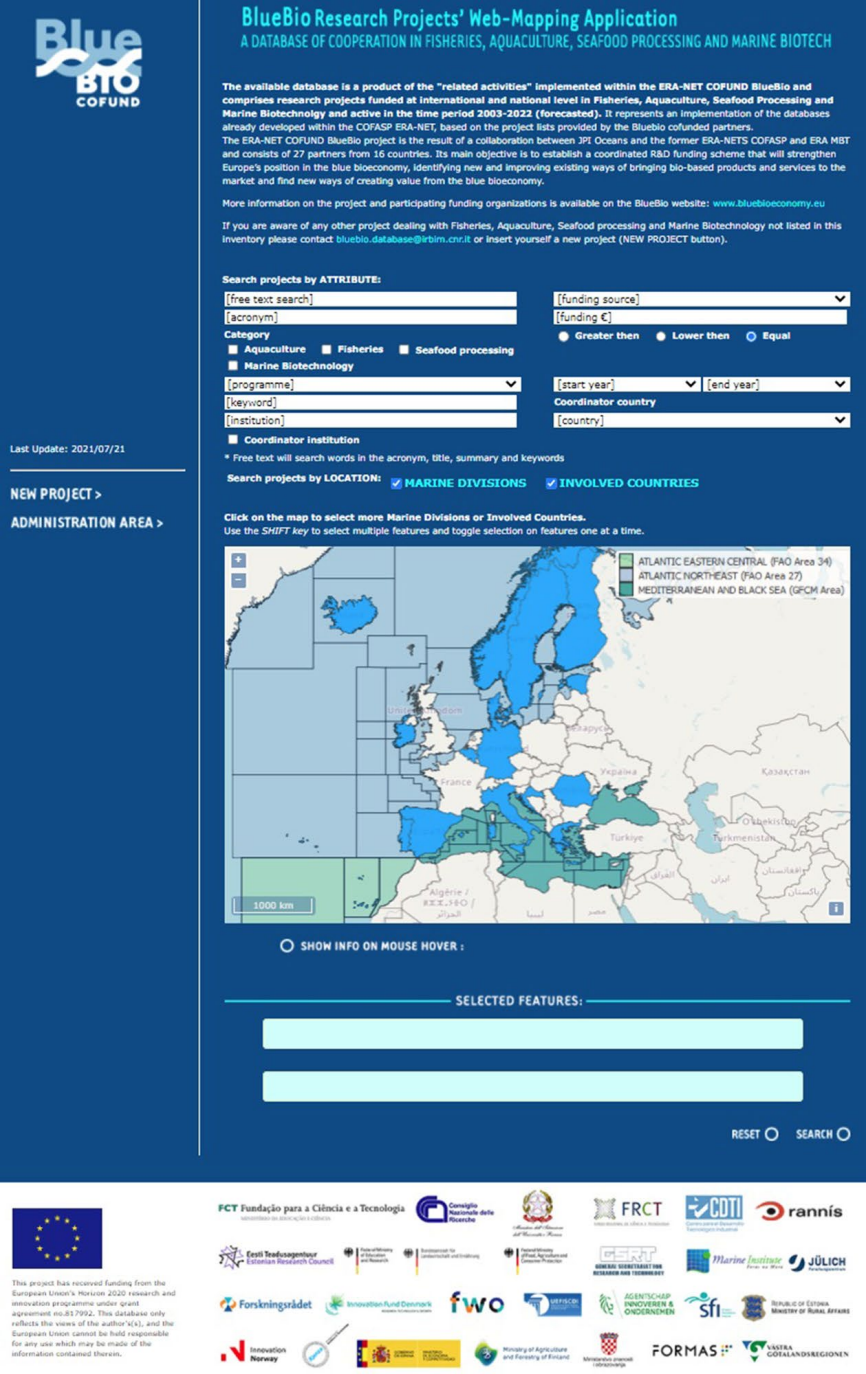
BlueBio online database: homepage to query data by attribute (e.g., category, programme, keyword, institution) and by location (selecting marine divisions or involved countries on the map), and the “New project” link (sidebar at left, it allows users to submit new entries). Basemap credits: © OpenStreetMap contributors licensed under the CC BY-SA 2.0 licence.

Information is disseminated through a web mapping application available on the ERA-NET Cofund BlueBio project website (https://bluebioeconomy.eu/the-bluebio-projects-online-database/; Fig. 1). It allows for: (i) easy and visual search of all projects carried out on a specific issue and/or geographical area and (ii) collaborative work, as users were/are invited and enabled to contribute to the collection of the data and the improvement of its reliability and availability.

From a technical point of view, the web application “spatially enables” the PostgreSQL object-relational database by relying on OpenLayers 3 (User Interface map component), Apache Web Server, MapQUest map server and GeoJSON features of countries and marine divisions (Web Map/Feature Access). The baseline PostegreSQL database as well as the web mapping application are hosted at CNR-IRBIM of Ancona, Italy.

The data set reported here is derived from the baseline PostegreSQL database and covers projects active in the period 2003–2019. The baseline database is continuously updated and widened, and it is foreseen to store projects active in the period 2003–2022 by the end of the BlueBio project.

The data collection and publication represent an unprecedented, consistent and robust recognition of the research carried out in Fisheries, Aquaculture, Seafood Processing and Marine Biotechnology at EU and country level. Although it does not have the ambition to include the entire universe of funded projects, the BlueBio database surely represents a unique collection gathering information from international and national repositories, archives of research institutes as well as from individual researchers and research projects’ websites.

It gives a picture and a map of the main research topics targeted by research in the EU and of the funding resources devoted to them. This information can be used by a range of stakeholders, from policy makers to researchers and producers as it allows to:

- identify relevant gaps and overlaps in the research on Fisheries, Aquaculture, Seafood Processing and Marine Biotechnologies at national/international level;
- take the stock of available knowledge to support the development of future research programmes both at national, regional and EU level;
- provide suitable material to identify potential synergies among actors and networks for future research projects.

## Methods

### Data collection

The data collection made within the COFASP project was extended in ERA-NET Cofund BlueBio through 4 surveys (once a year from 2019 to 2022) and an in-depth web and database search and review. The latter was carried out by database administrators (CNR-IRBIM) on the EU projects’ websites, the websites of research institutes/universities as well as on those of national and international funding agencies.

Surveys consisted of circulating a questionnaire - in .xls file with predefined fields to fill - amongst information producers (i.e., project coordinators, national research funding agencies involved in BlueBio projects); several reminds were sent to increase the response rate. Each Bluebio partner was clearly in charge of collecting its own national projects, asking for its own networks of research institutions and making great effort to directly contact relevant and priority projects. The questionnaire (BlueBio_database_data collection questionnaire.xlsx) is made available through the unrestricted repository at figshare^11^.

Note that the collection process ultimately depended on the identified key national contacts/information providers and their level of engagement with COFASP (before) and BlueBio (after) network and partners.

Data collection made also use of anonymous users who were able to submit independent records by using the “New project” module of the developed webGIS (Fig. 1). For this purpose, the webGIS and its web-based module for data entry was promoted during several BlueBio project meetings.

### Data harmonisation

The harmonisation process involved refining and cross-validating the collected information to allow comparison and analysis. It was long and time consuming.

First, a content cleaning process took place whereby the grammar, spelling and format were checked (e.g., institutions were standardised and traced back to predefined institutional signatures). Then, each entry (both by anonymous web-users and interviewed partners) was cross-validated against all the available data sources (e.g., questionnaires, institutional projects’ database and project’s specific websites) and, if necessary, integrated and edited by administrators before it was stored in the database. This process is hereinafter referred to as data retrieval (Table 1 and Table 2).

**Table 1:** Standard textual information fields used by the BlueBio database.

Information field	Name	Description	Origin of the information
1. Acronym	*acronym*	Official project acronym	Questionnaire/Data retrieval
2. Category	*category*	“Fisheries”, “Aquaculture”, “Seafood processing” or “Marine Biotechnology” (allowing cross-cutting options)	DB administrator
3. Title	*title*	Official project title	Questionnaire/Data retrieval
4. Website	*website*	Project official site	Questionnaire/Data retrieval
5. Summary	*summary*	Brief description of the project	Questionnaire/Data retrieval
6. Funding source	*fkfunding_source*	“National”, “European”, “National-European”, “Not Available” or “Other”	DB administrator
7. Funding	*funding*	Total funding allocated to the project (in EUR)	Questionnaire/Data retrieval
8. Start year	*start_year*	Starting year of the project	Questionnaire/Data retrieval
9. End year	*end_year*	Ending year of the project	Questionnaire/Data retrieval
10. Programme	*fkprogramme*	Main programme providing funds to the project	Questionnaire/Data retrieval
11. Programme1	*fkprogramme1*	Specific programme(s) (level of detail 1) providing funds to the project	DB administrator
12. Programme2	*fkprogramme2*	Specific sub-programme(s) (level of detail 2) providing funds to the project	DB administrator
13. Keyword	*keyword*	Keyword(s) from a predefined list (see Online-only Table 1, allowing more than one option)	DB administrator

*DB: database.

**Data retrieval: search made by DB administrators comparing and integrating from different sources of information (e.g., institutional projects’ database and project’s specific websites).

For a better characterization of the projects, based on the action fields of the BlueBio project, new fields of information were added such as identification by research category and source of funding.

Four main research categories were considered: *Fisheries*, *Aquaculture*, *Seafood Processing* and *Marine Biotechnology*. The combination of 2 or more categories was also considered to characterise cross-cutting research projects.

According to the related supporting programmes and instruments of funding, each project was also assigned to one of the following funding sources*: National, European, European/National* and *Other*. The former includes those projects that were exclusively funded within national programmes or instruments of funding (e.g., the Italian National Research Programme and Projects of Relevant National Interest), while the second includes EU Framework Programmes for Research and Technological Development/Research and Innovation (FP4 – FP8/HORIZON 2020) and EU funding programmes such as LIFE, COST or INTERREG. ERA-NET schemes and National Programmes supported by European Structural and Investment Funds are instead examples of what was labelled as *European/National*. Finally, projects falling out of the previous funding sources such as those funded by a consortium of Countries, international organisations, agencies or programmes not relying on EU funds were labelled as *Other* (e.g., Joint Programming Initiatives and Bilateral/Multilateral Cooperation Agreements).

An additional effort was made to harmonize the *programme* field. For example, the overarching funding programme was reported for each EU funded projects (e.g., FP4, FP5, Horizon 2020), the national projects were generalized as “National Programme”, while the projects cofounded trough the ERA-NET scheme were included in “International Cooperation”.

Regarding the projects’ budget, when necessary, it was translated into Euro using the exchange rate of the starting year of the project. Similarly, foreign projects’ abstracts were translated into English before they were stored in the database.

Last, to allow a better characterization of the projects and their easier search in the database, each project was associated with keywords taken from a list previously identified by database administrators (Online-only Table 1).

### Geographical extension

Projects were geographically allocated based on the marine area(s) where the research was carried out and the countries of the institutions involved. It allowed to highlight eventual differences between the European seas and/or countries.

Countries were directly linked to each institution, while projects were allocated into marine areas following these criteria:

- if the study area and/or case studies were clearly recognizable the project was associated with specific marine area(s);
- if the study area was not indicated but the project dealt with field experiments, the marine area of the coordinator country was used;
- if the study area was not indicated and the project did not deal with field experiments (e.g., laboratory genetic projects), the project was labelled as *Not associated with marine areas*. It was the case of those projects dealing on Aquaculture, Seafood Processing and Marine Biotechnology that were not specifically carried out at sea.

**Fig. 2:**
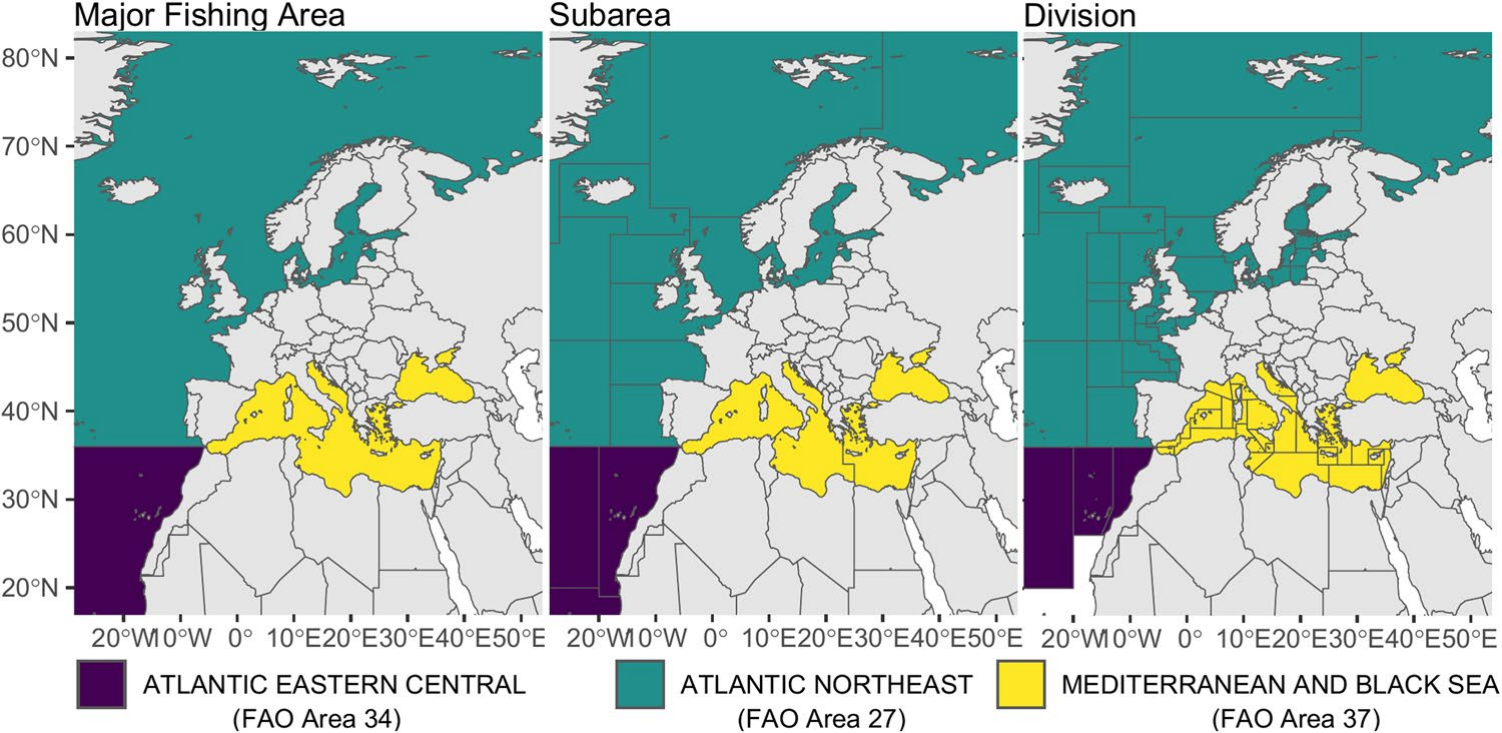
Major marine areas (left), subareas (central) and divisions (right).

The marine areas were identified following a hierarchical structure composed by 3 different levels of detail: Area, Subarea and Division. The identification of the Areas and Subareas was based on the Food and Agriculture Organization (FAO) Fishing Areas^12^: Atlantic Northeast (FAO Area 27), Atlantic Eastern Central (FAO Area 34) and Mediterranean and Black Sea (FAO Area 37). The FAO Fishing Divisions were also considered for the Atlantic Northeast and Atlantic Eastern Central, whereas the FAO-GFCM Geographical subareas (GSAs) were used for the Mediterranean and Black Sea. Overall, the 3 major Marine areas, Atlantic Northeast, Atlantic Eastern Central, Mediterranean and Black Sea were divided into 18 subareas and 75 divisions (Fig. 2, Online-only Table 2).

### Construction of the database

Tables 1, 2 summarise the information fields gathered through the collection and harmonisation process. Some were directly collected through questionnaires or by searching and comparing different sources of information such as institutional projects’ databases and project’s specific websites, while others were assigned by database administrators. Overall, 22 fields were associated with each record (project). Coordinator names and emails are stored in the database but not shared for privacy.

**Table 2:** Standard spatial information fields used by the BlueBio database.

Information field	Name	Description	Origin of the information
14. Coordinator Institution	*fkcoord_institution*	Name of the Institution acting as coordinator of the project	Questionnaire/Data retrieval
15. Coordinator Country	*country*	Name of the Country acting as coordinator of the project	Questionnaire/Data retrieval
16. Coordinator Continent	*continent*	Name of the Continent acting as coordinator of the project	Questionnaire/Data retrieval
17. Other Institutions involved	*institution*	Name(s) of the Institution(s) acting as partner(s) of the project (more than one)	Questionnaire/Data retrieval
18. Other Countries involved	*country*	Name(s) of the Country(s) acting as partner(s) of the project (more than one)	Questionnaire/Data retrieval
19. Other Continents involved	*continent*	Name(s) of the Continent(s) acting as partner(s) of the project (more than one)	Questionnaire/Data retrieval
20. Area	*area*	FAO major marine area (see Online-only Table 2, allowing more than one option)	Questionnaire/DB administrator
21. Sub-area	*subarea*	It depends on the Area (see Online-only Table 2, allowing more than one option)	Questionnaire/DB administrator
22. Division	*division*	It depends on the Sub-area (see Online-only Table 2, allowing more than one option)	Questionnaire/DB administrator

*Data retrieval: search made by DB administrators comparing and integrating from different sources of information (e.g., institutional projects’ database and project’s specific websites).

Program details (information fields: Programme1 and Programme2) are currently being harmonised by database administrators, and will be soon released in the next version of the data repository.

**Fig. 3:**
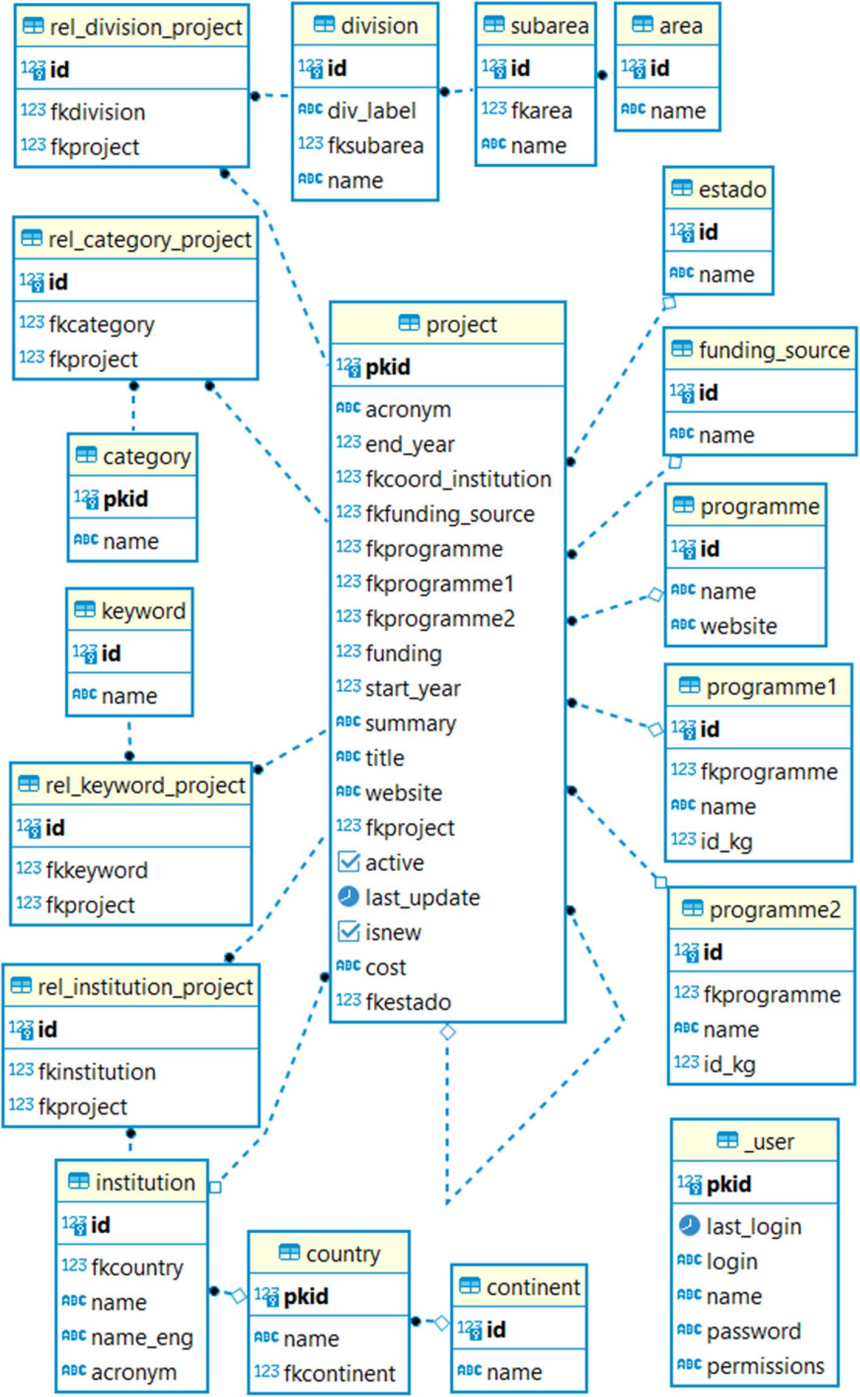
ER diagram showing the core BlueBio database tables.

The relational database was built in PostgreSQL and consists of a collection of tables that store interrelated data (Fig. 3). Each record is associated with a unique ID (e.g., pkid for each project) which allows creating relationships between tables. It was managed and maintained using different database management tools (e.g., pgAdmin).

### Data Records

Once subjected to the quality control procedures, the dataset presented in figshare^11^ (10.6084/m9.figshare.21507837.v3) is a copy of the database as it was on 28 August 2022. By this date, it consisted of 3,254 “georeferenced” records of national/international projects active in the time period 2003–2019. This dataset will be updated by the end of 2023 in figshare to reflect data additions (projects active in the period 2019‐2022) and updates (i.e., *programme1* and *programme2*).

The repository follows the FAIR principle of Findability, Accessibility, Interoperability and Reusability of data.

Some examples of information that can be drawn by the analysis of the data stored in the released BlueBio database are shown hereafter.

**Fig. 4:**
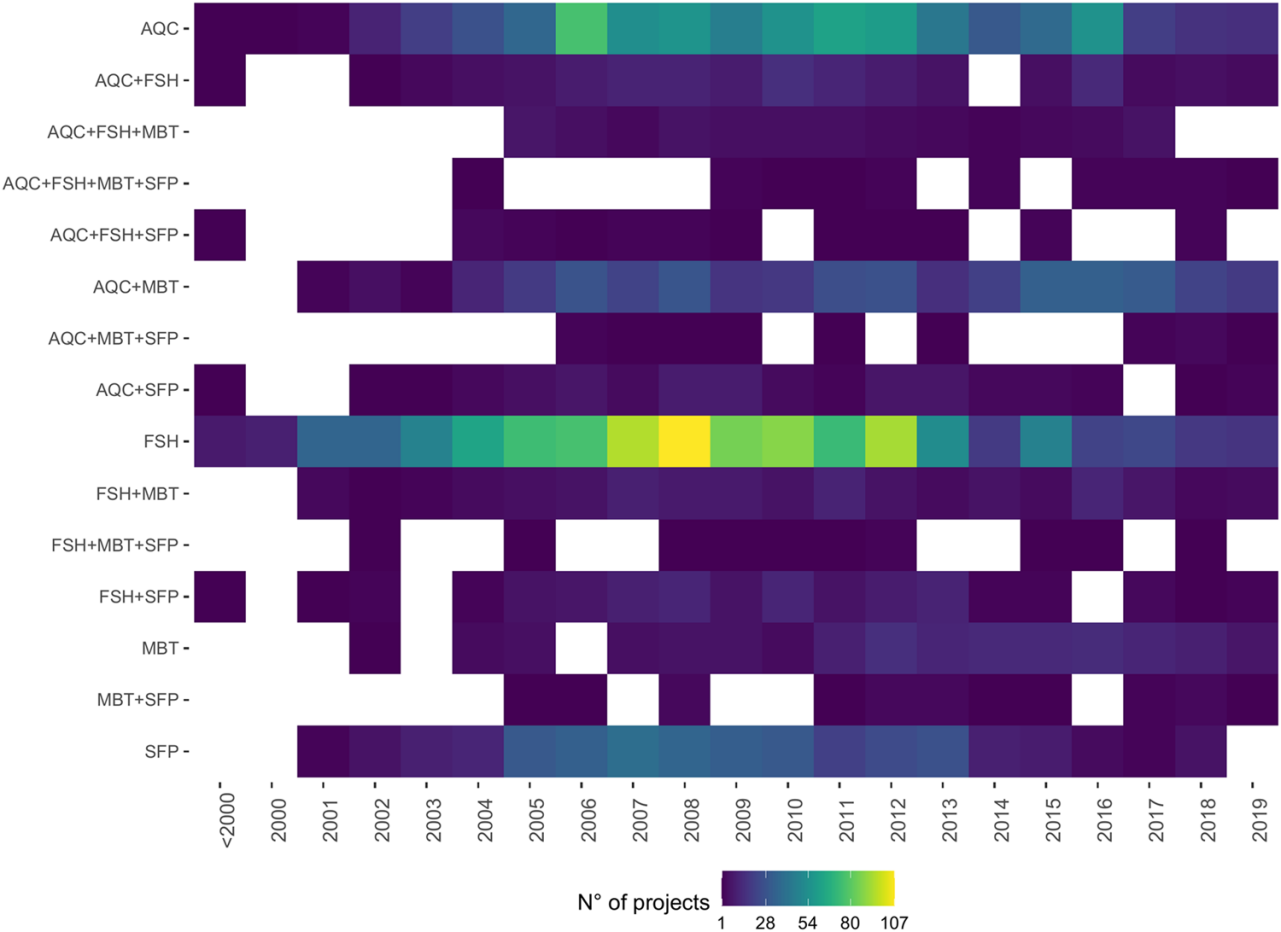
Number of projects by category and starting year (FSH: Fisheries, AQC: Aquaculture, SFP: Seafood Processing and MBT: Marine Biotechnology; including only projects for which starting year is available).

Most of the projects started in the period 2004–2017, and mainly focused on Fishery, Aquaculture and Seafood Processing (Fig. 4). Among the cross-cutting categories instead, Aquaculture & Marine Biotechnology was the most populated (12% of the projects), while all the others appeared poorly represented and accounted at most for 4% of the total (Aquaculture & Fisheries). Even though most of the 2-levels cross-cutting categories have been addressed since 2001–2002, the number of related projects remained rather low except for Aquaculture & Marine Biotechnology which slowly increased over time. The interdisciplinary projects addressing 3- and 4-level categories, instead, generally started later with a discontinuous trend and very low numbers.

**Fig. 5:**
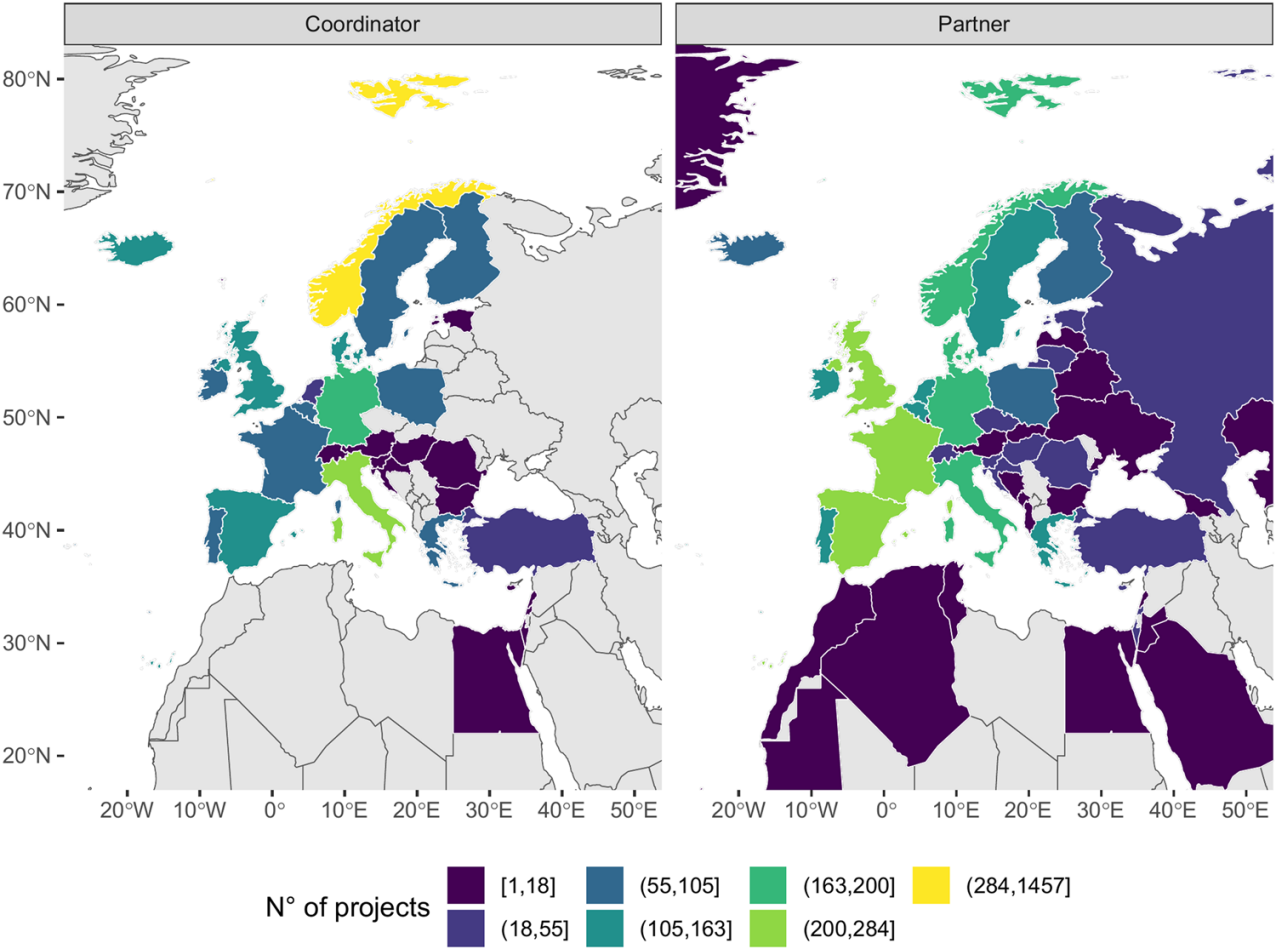
Number of projects by coordinator country (left) and involved country (right). Bounding box centred on Europe.

Overall, 26 out of the 96 countries involved were EU MS (including the United Kingdom as Brexit entered into force in 2020) and 58 non-EU countries. Norway dealt with the highest number of projects (1,649) followed by Italy, Spain, and the United Kingdom, which however participated in a far lower number of projects ranging from 427 to 467 (Fig. 5). Again, Norway coordinated the highest number of projects (more than 45% of the total universe of the database), followed by Italy (8%) and Germany (6%). On the other hand, Spain, the United Kingdom and France were involved in the highest number of projects as partners (8–9%; Fig. 5). A few countries (e.g., Germany, Poland and Finland) maintained a similar importance in the categorization both by coordinator and involved country, while others were only involved and never coordinated (e.g., Hungary and Lithuania).

**Fig. 6:**
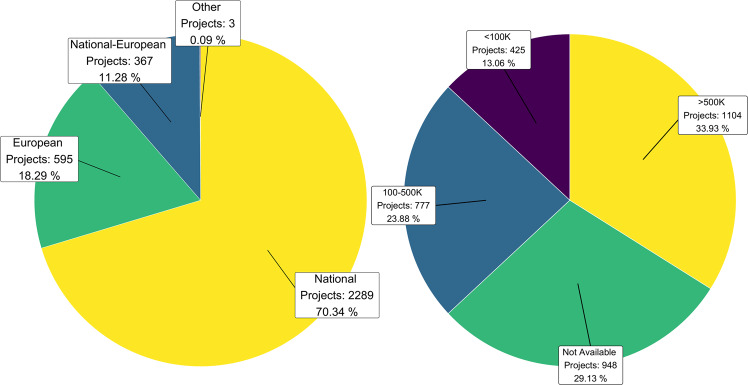
Number of projects by funding source (left) and by funding category (right).

The majority of the projects were funded at national level, while 18% by the European Commission (Fig. 6). The projects co-financed by European and National funds and those supported by Other funding sources accounted for 11% and 0.09%, respectively. Excluding projects for which the budget information was not available (948), 34% of the collected projects have a budget greater than 500 k€, 13% lower than 100 k€ and 24% between 100 k€ and 500 k€.

Projects with budgets >500 k€ represented 50% on average of the projects in each research category and around 90% of the total funding, while low budget projects (<100 k€), in general, did not exceed the 2% of the total funds of a research category.

Project’s budgets ranging between 100 and 500 k€ were quite important in almost all research categories exceeding 30% of the total projects, with the exception of Marine Biotechnology, Aquaculture & Fisheries, Aquaculture & Fisheries & Marine Biotechnology & Seafood Processing. However, they never exceeded 10% of the total funding of a research category.

**Fig. 7:**
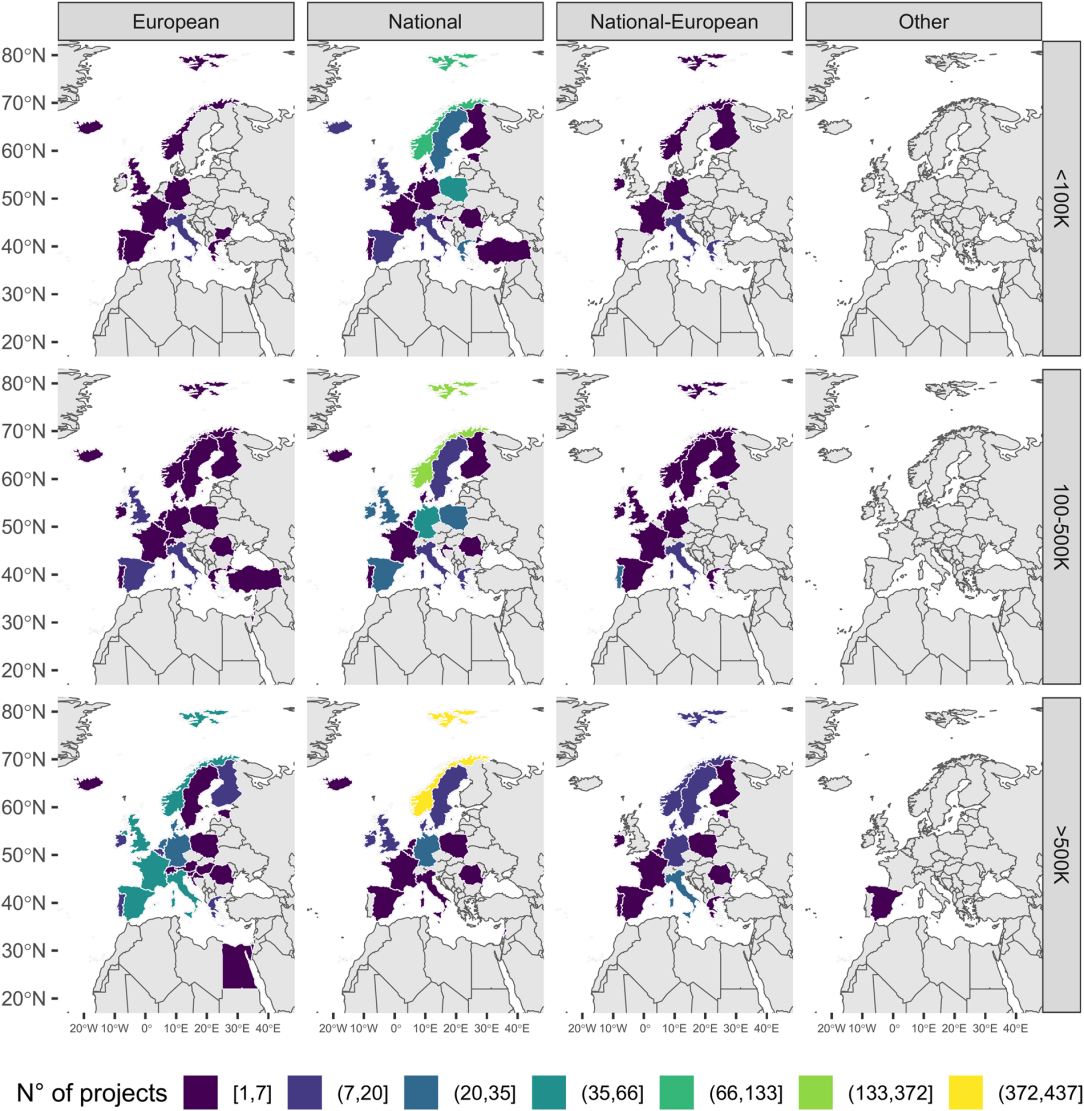
Number of projects by coordinator country, funding category (*European, National, National-European and Other*) and funding source (k€).

Bringing together information on funding sources and budget categories, data highlights that most of the projects coordinated by Norway had a budget >500 k€ and were funded by national programmes (Fig. 7). The same was in Italy, the United Kingdom, Spain and France but, in this case, most of the projects were funded by European funding programmes. On the contrary, in all countries the majority of projects with a budget less than 500 k€ were national, while the National-European funding programme financed mainly few projects with a budget >500 k€.

Deeper analysis of topics within each research category highlighted research priorities and needs as well as eventual differences among European marine areas and countries. Obviously, outcomes strongly depend on the identification of topics by users. With regards to Aquaculture, for example, Table 3 lists the 16 main topics identified, among which excel Aquaculture development and/or management, Animal welfare and/or health, Animal feed, and Engineering.

**Table 3:** Topics identified within the Aquaculture category, with related number of projects and total budget (Mln €).

Topic	n. projects	Mln € (n. projects*)
Algae	34	14,32 (25)
Animal feed	157	69,74 (125)
Animal welfare and/or health	229	78,14 (174)
Aquaculture development and/or management	315	222,39 (227)
Biology and/or Ecology	58	10,6 (40)
Cephalopods	2	0 (0)
Economy	19	11,34 (13)
Engineering	144	66,04 (108)
Environmental impact	66	30,12 (48)
Fish	412	114,68 (305)
Human food and/or Seafood quality and/or Safety	29	6,58 (22)
Impacts	20	5,65 (16)
Land-based aquaculture	80	19,57 (59)
Open-sea aquaculture	122	35,7 (93)
Other organisms	8	1,71 (6)
Shellfish	71	21,65 (48)

*Number of projects by topic with available budget information.

**Fig. 8:**
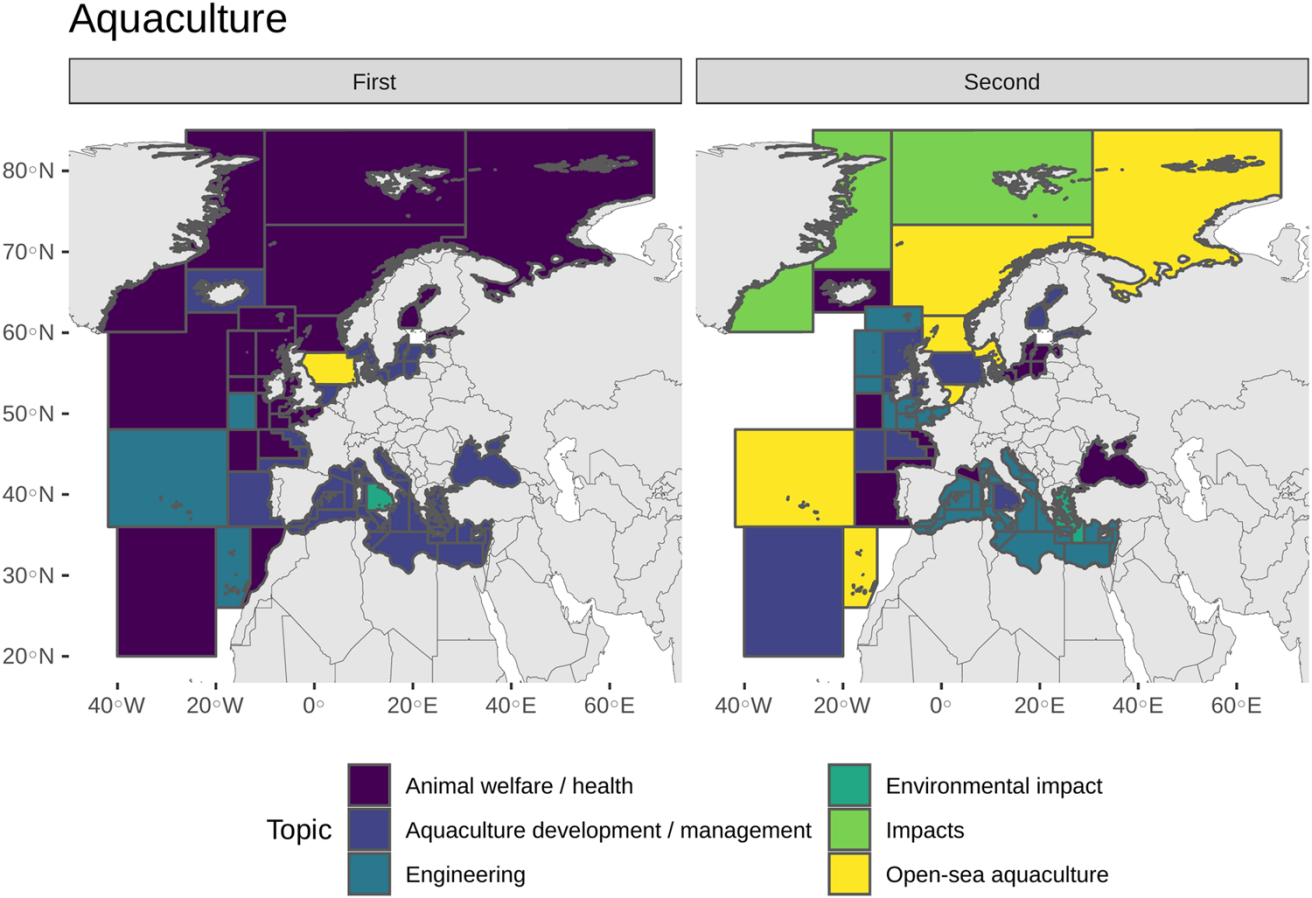
Aquaculture category and related first and second main thematic topics (left and right, respectively) by marine division.

However, Animal welfare and/or health - mainly consisting in the development of farming systems to improve productivity and product quality by increasing welfare and lower the risks of diseases in the farmed species - seemed to be the priority almost everywhere in the Atlantic Northeast (FAO Area 27) and in the coastal waters of Morocco (FAO Area 34) (Fig. 8). Other relevant issues in these areas were related to open-sea aquaculture and the evaluation of impacts induced on farmed species by other human activities or environmental stressors (e.g., climate change, ocean acidification, algal toxins). In the remaining areas of the Atlantic Northeast (Iceland Grounds, central and southern Baltic Sea, Bay of Biscay, Portuguese waters) and in the Mediterranean and Black Sea (FAO Area 37), instead, the most addressed topic was Aquaculture development and/or management, which comprises either projects aimed to push the sector’s production by implementing larval rearing for already farmed and new species and projects dealing with the management of aquaculture and its sustainability, including marine spatial planning. Additional relevant topics are Engineering (i.e., technological development of aquaculture systems both at open sea and land), and Animal welfare and/or health, the latter limited to the Black Sea. In contrast, very few projects dealt on the implementation of integrated multitrophic aquaculture and offshore integrated platforms. Also, the assessment of impacts induced by aquaculture on the marine ecosystem did not appear a priority. Fish appeared the most investigated taxonomic group followed by shellfish (molluscs and crustaceans) and algae. A very low number of projects targeted other low trophic organisms, e.g., ascidians, sea cucumbers, jellyfish, krill.

## Technical Validation

The data collected through the questionnaires were checked against additional sources such as, for example, CORDIS website for EU-funded projects and single project’s websites. This process, in parallel with the data harmonisation, was manual and very time consuming.

**Fig. 9:**
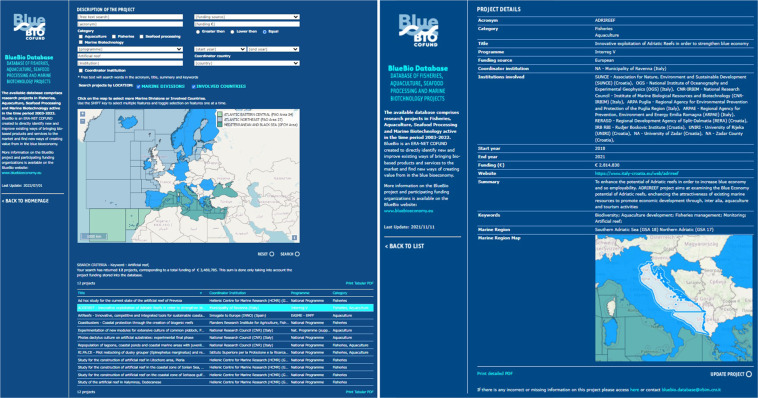
BlueBio online database: Results page (left) with the list of projects resulted by the set search criteria, and Individual Result page (right) with the project’s details and the lower right button to submit project updates. Basemap credits: © OpenStreetMap contributors licensed under the CC BY-SA 2.0 licence.

The webGIS itself was developed and used to validate the dataset, as users could submit their own editing through the dedicated update module (Fig. 9) while querying the database. Each proposed online update, as well as each new entry, needed to be validated (through a cross-check among different databases and project’s websites) and approved by database administrators prior to becoming permanent and available on-line. When updating a pre-existing project, online users can update the information already reported in the database and/or add new ones in the empty fields.

Less useful was the dedicated email address, as only a few users reported to the DB administrators with incorrect or missing information.

In the overall, all the process (data collection, verification, harmonization) required more than 8000 hours.

## Usage Notes

The dataset was released under the Creative Commons Attribution licence (CC-BY, v. 4.0, https://creativecommons.org/licenses/by/4.0/deed.it, last access: 3 May 2021). It is available as SQL dump (backup_BlueBio_db_28082022.sql) and in a more user-friendly CSV format (Bluebio_db_28082022.csv), with 3,254 rows and sensible column headings (as described in the readme file in figshare^11^).

Data are versioned, as they are updated periodically (in this article, results from the dataset V3 of August 2022 are reported). While waiting for the next update of the repository, the web mapping application allows users to print in pdf filtered list of projects or single project details as soon as they are stored.

The dataset can be helpful to various end users, from policy makers to researchers and producers. Providing information on research projects – including those that could be difficult to find (e.g., national projects, bilateral international projects) - it contributes to exchange knowledge and/or technology hence supporting “the sharing and reuse of research data” and open science. Moreover, it represents a tool to support the EU’s Research & Innovation policy aimed to strengthen the scientific and technological development of the Union and foster its competitiveness, including in its industry, in a context of sustainable growth^12^.

For instance, scientists who are going to draft research proposals could examine the data to be informed on the state-of-art in the field of interest in terms of topics addressed and geographical areas covered in Europe. It would allow them to contextualise and make their research innovative, e.g., they could verify which are the most targeted species or the most updated technological developments in the aquaculture sector.

Searching on the dataset also allows scientists to create networks with other research institutes and/or universities and private companies working in the same field, thus encouraging sharing of knowledge and known-how, benchmarking and cooperation among different actors on strategic research issues, and improving an efficient use of resources among projects. On the other hand, private companies could query the dataset to search for research institutes able to support their R&D team in the development of new technologies and products.

The dataset could be also used by policy makers to identify potential experts to be involved in scientific advisory bodies called to provide advice and support decisional processes, e.g. Scientific, Technical and Economic Committee for Fisheries (STECF), International Council for the Exploration of the Sea (ICES) and General Fisheries Commission for the Mediterranean (GFCM). Moreover, it could be key to verify available information on short-term needs and to identify gaps to be addressed in the short-time through scientific advice studies (e.g., call for tenders and call for proposals) or in the long-term through research projects supported under national/international research framework programmes.

In spite of its large coverage in terms of projects funded under different funding programmes, geographical areas and countries, the dataset presents however some limitations to be taken into consideration by users. First, it mainly covers projects involving the countries participating in the COFASP ERA-NET and the ERA-NET COFUND BlueBio (i.e., Belgium, Croatia, Denmark, Estonia, Finland, France, Germany, Greece, Iceland, Ireland, Italy, Latvija, Malta, Netherland, Norway, Portugal, Romania, Spain, Sweden, Turkey and the United Kingdom). Second, it was not possible to update the database in respect to the national projects funded by France, Netherland, United Kingdom and Turkey because these countries joined the COFASP ERA-NET but not the ERANET COFUND BlueBio.

To geographically reallocate projects, shapefiles of marine areas (FAO Fishing Divisions and FAO-GFCM GSAs) can be downloaded at: https://www.fao.org/fishery/area/search/en^13^ and https://www.fao.org/gfcm/data/maps/gsas/en/; while vector data of world countries are available in several public domain map datasets such as Natural Earth (https://www.naturalearthdata.com/).

## Data Availability

From the BlueBio PostgreSQL database, the .CSV dataset was extracted via queries through the free software environment R^14^, using the *dbConnect* function from the RPostgreSQL package^15^, a database interface and ‘PostgreSQL’ driver for R. The related R code (dbconnect_csv.R) is freely available through figshare^11^. The maps in Figs. 2, 5, 7, 8 were created using several R packages like *tidyverse*^16^ for data handling, *rgdal*^17^ package providing bindings to the “Geospatial Data Abstraction Library”, *sf package*, a standardised way of encoding spatial vector data in R and the package *ggplot* for graphical visualisation. The R code (scidata_workflow.R) and the additional data layer for the creation of all the reported figures and summary statistics are deposited in the figshare^11^ public repository. This could facilitate data re-use and analysis.

## Online-only Tables

**Online-only Table 1:** List of Keywords.

Abalone	Cephalopod	Flatfish	Metabolites	Sea ranching
Acoustic survey	Certification	Floating structures	Metagenomic	Sea turtles
Algae	Cetaceans	Flounder	Microbial communities	Sea urchin
Algal toxins	Changeable nassa	Food products	Microbiome	Seabass
Alien species	Clam	Food quality	Microplastics	Sea bream
Amberjacks	Climate change	Food safety	Mollusc	Seafood
Anchovy	Clown fish	Food web	Monitoring	Seagrass
Anglerfish	Cobia	Fuel consumption	MPA	Seals
Animal feed	Cod	Gastropod	Mussel	Sea star
Animal welfare	Common piddock	Gear selectivity	Nursery area	Seed production
Anthropic activity	Corals	Gear technology	Nutraceutical substances	Selective breeding
Antifouling	Crab	Genetic	Octopus	Sharks
Antimicrobials	Crustacean	Genomic and gene mining	Offshore platforms	Shellfish
Aquaculture development	Crustacean fisheries	Genomic sequencing	Offshore renewable energy	Shi Drum
Aquaculture diversification	Cuttlefish	Germ cell-free animals	Open sea aquaculture	Shrimp
Aquaculture industry	Diagnostic application	Gillnets	Oyster	Slaughtering systems
Aquaculture management	Diets	Grey mullet	Packaging	Small scale fisheries
Aquaponics	Discard	Grooved carpet shell	Parasite	Sole
Artic charr	Disease	Growth rate	Passive gears	Spatial planning
Artificial reef	Dissemination	Guidelines	Physical disturbance	Spawning area
Ascidian	Dolphins	Habitat mapping	Plaice	Sponge
Bacteria	Drift nets	Habitat enhancement	Policy	Sprat
Bacteriocins	Drug discovery	Haddock	Pollution	Squid
Barnacle	Dusky grouper	Hake	Population dynamic	Stock
Benthic communities	Echinoderm	Halibut	Population structure	Stock assessment
Bioactive compounds	Economy	Herring	Probiotics	Stock enhancement
Biocatalyses	Ecosystem approach	Houting	Process efficiency	Storage
Biodegradation	Eel	Human food	Product development	Striped venus
Biodiversity	Engineering	Human health	Production	Sturgeon
Biofilm	Environmental impact	Impacts	Production management	Sustainability
Biogenic reefs	Escapes	Indicators	Protein	Swordfish
Biofouling	Estuarine fisheries	Integrated management	Protein source	Tagging
Biofuel	Exploitation	Integrated multi-trophic aquaculture	Protocol	Toxic substances
Biology	Extreme enzymes	Jellyfish	Prototype	Toxins
Biomaterial	Feed composition	Krill	Purse seine	Traceability
Biomass	Feed quality	Labelling	Quality	Trammel nets
Biomimicry	Fish	Land-based aquaculture	Queen conch	Traps
Biopolymer	Fish aggregating device	Landing	Quota regulation	Trawling
Bioprospecting	Fish biology	Larval development	Ray	Trout
Biorefinery	Fish habitat	Larval dispersion	Razor clam	Tuna
Bioremediation	Fish health	Larval mortality	Recirculating systems	Turbot
Biosensors	Fish meal replacement	Larval quality	Recreational fisheries	Vaccines development
Biotechnology	Fish oil replacement	Larval rearing	Recruitment	Value chain
Bivalve	Fish products	Life cycle	Red mullet	VMS data
Black scabbardfish	Fish reproduction	Lobster	Red porgy	Waste management
Blue economy	Fish stocks	Logbook	Redfish	Waste water
Blue growth	Fisheries development	Longline fishing	Restocking	Wastes
Blue whiting	Fisheries management	Longline systems	Restoration	Water management
Brill	Fisheries research	Lump fish	Risk assessment	Waste valorization
Broodstocks	Fishery policy	Mackerel	Saithe	Water quality
Bycatch	Fishing effort	Manila clam	Salmon	Whale
Byproducts	Fishing fleets	Mapping	Sand steenbras	Whitefish
Cage aquaculture	Fishing industry	Marine enzymes	Sandeel	Wild animals
Capture-based aquaculture	Fishing mortality	Marine litter	Sardine	Wrasse
Carp	Fishing technology	Market	Scallop	Wreckfish
Catch	Fishing vessels	Meagre	Sea cucumber	Zooplankton

**Online-only Table 2:** List of Marine Areas.

AREA	SUBAREA	DIVISION
ATLANTIC EASTERN CENTRAL (FAO Area 34)	Northern Coastal	Canarias and Madeira Islands (34.1.2)
		Morocco coastal (34.1.1)
	Northern Oceanic	Northern Oceanic (34.2)
ATLANTIC NORTHEAST (FAO Area 27)	Azores Grounds Azores	Azores Grounds (27.Xa, 27.Xb)
	Barents Sea	Barents Sea (27.I)
	Bay of Biscay	Bay of Biscay Central (27.VIIIb)
		Bay of Biscay North (27.VIIIa)
		Bay of Biscay offshore (27.VIIId)
		Bay of Biscay Southern (27.VIIIc)
		West of Bay of Biscay (27.VIIIe)
	East Greenland	Northeast Greenland (27.XIVa)
		Southeast Greenland (27.XIVb)
	Iceland and Faroes Grounds	Faroes Grounds (27.Vb)
		Iceland Grounds (27.Va)
	Irish Sea, West of Ireland, Porcupine Bank, English Channel, Bristol Channel, Celtic Sea, Southwest of Ireland	Bristol Channel (27.VIIf) (27.IIId.25)
		Celtic Sea North (27.VIIg)
		Celtic Sea South (27.VIIh)
		Eastern English Channel (27.VIId)
		Irish Sea (27.VIIa)
		Porcupine Bank (27.VIIc)
		Southwest of Ireland-East (27.VIIj)
		Southwest of Ireland-West (27.VIIk)
		West of Ireland (27.VIIb)
		Western English Channel (27.VIIe)
	North of Azores	North of Azores (27.XIIa, 27.XIIb, 27.XIIc)
	North Sea	Central North Sea (27.IVb)
		Northern North Sea (27.IVa)
		Southern North Sea (27.IVc)
	SeaNorwegian Sea, Spitzbergen and Bear Island	Norwegian Sea (27.IIa)
		Spitzbergen and Bear Island (27.IIb)
	Portuguese Waters	Portuguese Waters (27.IXa, 27.IXb)
	Rockall, Northwest Coast of Scotland and North Ireland	Northwest Coast of Scotland and North Rockall (27.VIb)
	Skagerrak, Kattegat, Sound, Belt Sea and Baltic Sea	Archipelago Sea (27.IIId.29)
		Baltic West of Bornholm (27.IIId.24)t
		Bothnian Bay (27.IIId.31)
		Bothnian Sea (27.IIId.30)
		East of Gotland or Gulf of Riga (27.IIId.28)
		Gulf of Finland (27.IIId.32)
		Skagerrak, Kattegat (27.IIIa)
		Sound, Belt Sea (27.IIIb, 27.IIIc)
		Southern Central Baltic-East (27.IIId.26)
		Southern Central Baltic-West (27.IIId.25)
		West of Gotland (27.IIId.27)
MEDITERRANEAN AND BLACK SEA (GFCM Area)	Western Mediterranean (Subarea 37.1)	Northern Alboran Sea (GSA 1)
		Alboran Island (GSA 2)
		Southern Alboran Sea (GSA 3)
		Algeria (GSA 4)
		Balearic Island (GSA 5)
		Northern Spain (GSA 6)
		Sardinia West (GSA 11.1)
		Gulf of Lions (GSA 7)
		Corsica Island (GSA 8)
		Ligurian and North Tyrrhenian Sea (GSA 9)
		South Tyrrhenian Sea (GSA 10)
		Sardinia East (GSA 11.2)
		Northern Tunisia (GSA 12)
	Central Mediterranean (Subarea 37.2)	Northern Adriatic Sea (GSA 17)
		Southern Adriatic Sea (GSA 18)
		Gulf of Hammamet (GSA 13)
		Gulf of Gabes (GSA 14)
		Malta Island (GSA 15)
		South of Sicily (GSA 16)
		Western Ionian Sea (GSA 19)
		Eastern Ionian Sea (GSA 20)
		Southern Ionian Sea (GSA 21)
	Eastern Mediterranean (Subarea 37.3)	Aegean Sea (GSA 22)
		Crete Island (GSA 23)
		North Levant (GSA 24)
		Cyprus Island (GSA 25)
		South Levant (GSA 26)
		Levant (GSA 27)
	Black Sea (Subarea 37.4)	Marmara Sea (GSA 28)
		Black Sea (GSA 29)
		Azov Sea (GSA 30)

## References

[1] Projects & results | CORDIS | European Commission. https://cordis.europa.eu/projects/it. (Accessed: 31 July 2022).

[2] Keep.eu. https://keep.eu/. (Accessed: 31 July 2022).

[3] Matís. https://matis.is/en/rannsoknir-og-nyskopun/rannsoknarverkefni/. (Accessed: 31 July 2022).

[4] National Marine Fisheries Research Institute. https://mir.gdynia.pl/?lang=en. (Accessed: 31 July 2022).

[5] FHF Prosjektbasen. https://www.fhf.no/prosjekter/prosjektbasen/. (Accessed: 31 July 2022).

[6] FCT - Fundação para a Ciência e a Tecnologia. https://www.fct.pt/. (Accessed: 31 July 2022).

[7] BlueBio Cofund. *BlueBio Cofund* https://bluebioeconomy.eu/. (Accessed: 05 August 2022).

[8] *Strategic research agenda for Fisheries, Aquaculture and Seafood Processing. COFASP ERA-net*. **40** https://cofasp.bluebioeconomy.eu/wp-content/uploads/2020/01/COFASP_SRA_for_web_compressed.pdf (2016).

[9] De Raedemaecker, F. *ERA-MBT Final dissemination report. Marine Biotechnology ERA-NET*. **29** http://www.marinebiotech.eu/outreach-material (2017).

[10] van Hoof L (2019). Food from the ocean; towards a research agenda for sustainable use of our oceans’ natural resources. Marine Policy.

[11] Spagnolo A (2022). Figshare.

[12] Kastrinos N, Weber KM (2020). Sustainable development goals in the research and innovation policy of the European Union. Technological Forecasting and Social Change.

[13] FAO. FAO Statistical Areas for Fishery Purposes. In: FAO Fisheries and Aquaculture Department [online]. Rome. https://www.fao.org/fishery/area/search/en (2020).

[14] R Core Team. *R: A Language and Environment for Statistical Computing*. (R Foundation for Statistical Computing, 2022).

[15] Conway, J., Eddelbuettel, D., Nishiyama, T., Prayaga, S. K. & Tiffin, N. RPostgreSQL: R Interface to the ‘PostgreSQL’ Database System. https://CRAN.R-project.org/package=RPostgreSQL (2022).

[16] Wickham, H. tidyverse: Easily Install and Load the ‘Tidyverse’. https://www.tidyverse.org (2022).

[17] Bivand, R. *et al*. rgdal: Bindings for the ‘Geospatial’ Data Abstraction Library. https://CRAN.R-project.org/package=rgdal (2022).

